# SynNotch CAR-T cell, when synthetic biology and immunology meet again

**DOI:** 10.3389/fimmu.2025.1545270

**Published:** 2025-04-16

**Authors:** Mohsen Shirzadian, Sepideh Moori, Reza Rabbani, Fatemeh Rahbarizadeh

**Affiliations:** ^1^ Department of Molecular Genetics, Faculty of Biological Sciences, Tarbiat Modares University, Tehran, Iran; ^2^ Department of Medical Biotechnology, Faculty of Medical Sciences, Tarbiat Modares University, Tehran, Iran; ^3^ Department of Stem Cell Technology and Tissue Engineering, Faculty of Interdisciplinary Science and Technology, Tarbiat Modares University, Tehran, Iran

**Keywords:** synNotch receptor, CAR-T cell therapy, synthetic biology, immunotherapy, cancer, molecular logic-gates

## Abstract

Cancer immunotherapy has been transformed by chimeric antigen receptor (CAR) T-cell treatment, which has shown groundbreaking results in hematological malignancies. However, its application in solid tumors remains a formidable challenge due to immune evasion, tumor heterogeneity, and safety concerns arising from off-target effects. A long-standing effort in this field has been the development of synthetic receptors to create new signaling pathways and rewire immune cells for the specific targeting of cancer cells, particularly in cell-based immunotherapy. This field has undergone a paradigm shift with the introduction of synthetic Notch (synNotch) receptors, which offer a highly versatile signaling platform modeled after natural receptor-ligand interactions. By functioning as molecular logic gates, synNotch receptors enable precise, multi-antigen regulation of T-cell activation, paving the way for enhanced specificity and control. This review explores the revolutionary integration of synNotch systems with CAR T-cell therapy, emphasizing cutting-edge strategies to overcome the inherent limitations of traditional approaches. We delve into the mechanisms of synNotch receptor design, focusing on their ability to discriminate between cancerous and normal cells through spatiotemporally controlled gene expression. Additionally, we highlight recent advancements to improve therapeutic efficacy, safety, and adaptability in treating solid tumors. This study highlights the potential of synNotch-based CAR-T cells to transform the field of targeted cancer therapy by resolving present challenges and shedding light on potential future paths.

## 1 Background

Synthetic biology, a field that merges engineering principles with biology, has revolutionized the development of novel therapeutic strategies. By designing and constructing new biological systems, researchers are able to engineer cells with enhanced functions, such as the ability to target and destroy cancer cells. Synthetic receptors, a key component of synthetic biology, enable the creation of chimeric proteins that combine the specificity of natural receptors with the effector functions of immune cells. These engineered receptors can be used to reprogram immune cells to recognize and eliminate cancer cells more effectively. Immunotherapy harnesses the power of the immune system to fight cancer, employing synthetic receptors to target tumor cells at various sites (1). Immunotherapy primarily aims to enhance the immune system by modulating the immunological microenvironment, enabling immune cells to target and eliminate tumor cells at critical junctures (2).

In recent years, immunotherapy has proved to be effective in treating cancer by manipulating the immune system. Adoptive cell therapy (ACT) is a brilliant and efficient method of immunotherapy, as it offers more personalized and targeted treatments among the various forms of immunotherapy (3).

The chimeric antigen receptor (CAR) T cells, which are a subset of ACT, are a frontier with unique outcomes in cancer therapy. Thus far, the FDA has approved six pharmaceuticals that utilize CAR T against hematological malignancies: Kymriah, Yescarta, Tecartus, Breyanzi, Abecma, and Carvykti (4). Chimeric Antigen Receptors (CARs) are special receptors that have a part that recognizes antigens outside of cells and a part that sends signals inside of cells. CARs enable T cells to target tumor-associated antigens (TAAs) without relying on MHC (5).

CARs can also target antigens that elicit a weak T-cell response or have a low abundance of specific T cells, such as certain tumor-associated peptide–MHC complexes (6, 7).

While CAR-T cell therapy has shown great promise for various blood cancers, it still faces significant challenges that need to be addressed, especially in treating solid tumors. These challenges include issues such as the scarcity of tumor-specific antigens (TSA) in many solid tumors, which means that CAR-T cells may also react with normal tissues, leading to on-target off-tumor (OTOT) effects and potential severe damage to healthy tissues. In B cell malignancies, the CD19 antigen is often targeted to eliminate abnormal B cells. Despite the loss of normal B cells, patients can usually tolerate B cell aplasia, resulting in promising outcomes for CAR-T cell therapy in these cancers. Another limitation is the inhibitory conditions within the tumor microenvironment (TME), which impede the activation and proliferation of infiltrating cells. Additionally, the limited trafficking of CAR-T cells to tumor cells, T-cell exhaustion, and cytokine release syndrome (CRS) present further challenges. Due to the heterogeneity of solid tumors and their varied antigen expression, designing a CAR-T cell that targets a single antigen may lead to tumor escape and relapse after initial remission (8).

One of the reasons for failure in immunotherapy methods, which was identified years later, was that tumor cells can change or reduce the expression of their antigens by mechanisms such as genetic alteration (9–11), epigenetic modification (12), clonal selection (13), and antigen shedding (14) and, as a result, escape from the immune system. This intelligence of cancer cells is still a major obstacle in cancer treatment, and for this reason, the selection of the target antigen is one of the bottlenecks for the success or failure of an immunotherapy drug against cancer (15).

**Figure 1:**
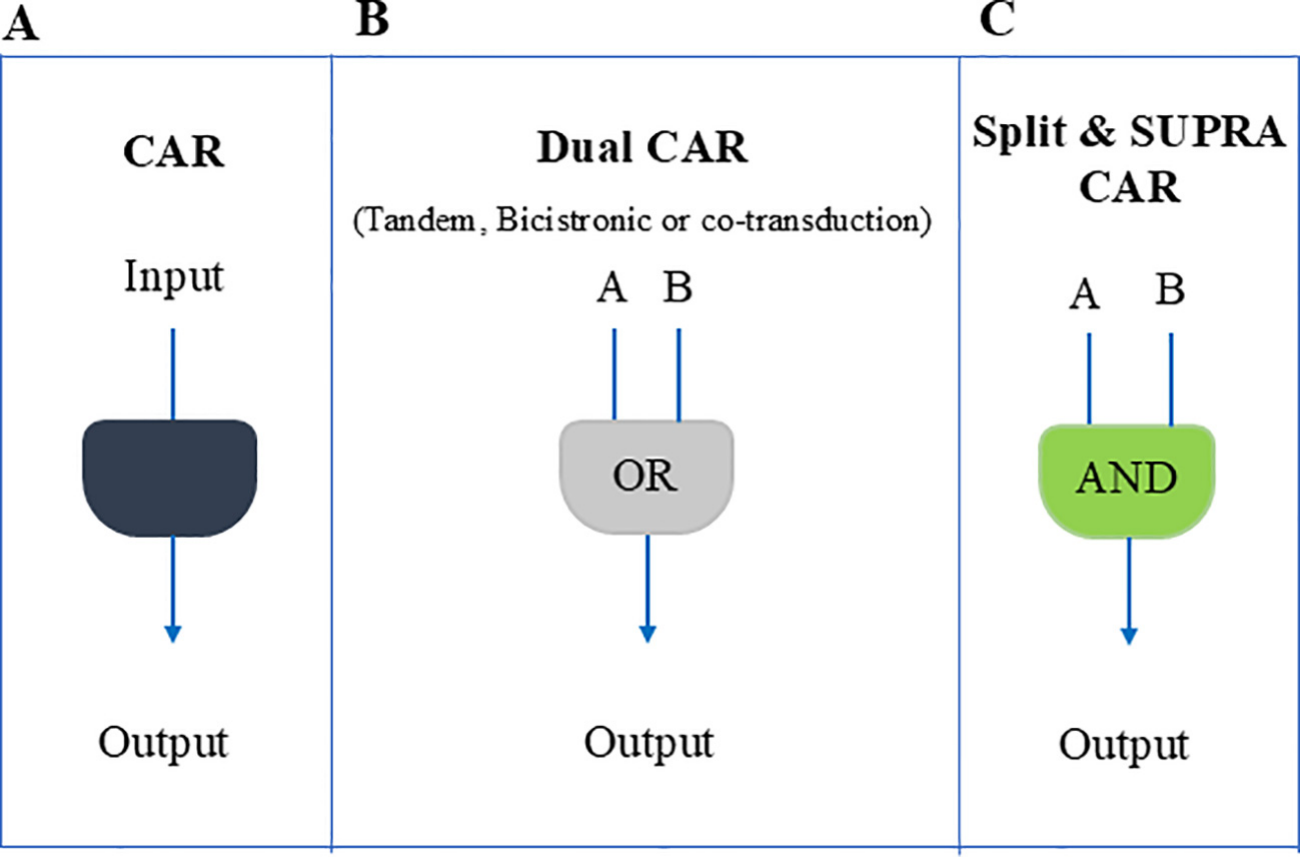
Schematic overview of different CAR-T cell design strategies used to counter antigen loss and enhance tumor specificity. **(A)** A conventional single CAR or TCR that recognizes one antigen. Although simpler, tumors may escape if that antigen is downregulated. **(B)** A dual CAR approach (tandem, bicistronic, or via co-transduction), functioning largely as an OR gate, so T cells can respond if at least one of two antigens is present. This helps reduce immune escape due to antigen downregulation but can still allow off-target effects. **(C)** A split or SUPRA CAR system that requires engagement of two separate receptors (an AND gate) for full activation, thereby enhancing specificity and minimizing unintended toxicity.

In the past decade, researchers have implemented numerous strategies to mitigate the adverse effects of CAR-T cell therapy and enhance its effectiveness. Such approaches involve the use of tandem CARs (16–18), T cells that express two distinct CARs (bicistronic CAR-T cells) (19, 20), co-transduction of T cells with two vectors encoding the two separate CARs (21), split CAR (22), or split, universal, and programmable (SUPRA) CAR system (23), which can potentially minimize off-target effects through a dual antigen recognition circuit. (**Figures 1A-C**).

Despite advancements through the introduction of logic-gated CAR-T cells, challenges such as downstream signaling kinetic match, complex construct design, and expression equality limit its clinical application.

In 2016, Roybal et al. introduced a novel concept that involves utilizing a synthetic Notch receptor (SynNotch) alongside CARs. This innovative approach allows for the identification of multiple antigens and mitigates downstream signaling interference (24). SynNotch technology could potentially overcome some of the limitations of long-established AND-gated CAR-T cells due to its valuable advantages.

In this review, we will examine synNotch CAR-T cells, exploring their potential in treating various cancers, strategies to enhance their efficacy, and solutions to overcome current limitations.

## 2 Synthetic notch receptor: design and function

In this section, we will examine the molecular components of synNotch constructions that contribute to the synNotch CAR-T cell end product. Our focus will be exclusively directed toward synNotch, as there are a multitude of exceptional articles that provide a comprehensive examination of CAR construction. We recommend reading the articles by Marzieh Mazinani et al. (25) and Sonia Guedan et al. (26) for further insights into CAR construction.

### 2.1 SynNotch construct components

In multicellular eukaryotes, the Notch signaling pathway is a highly conserved signaling cascade that regulates homeostasis, morphogenesis, and spatial patterning in both embryonic and adult tissues (27, 28). Notch proteins regulate tissue homeostasis through receptor-ligand interactions on neighboring cells (29, 30). Notch receptors (N1–N4) and ligands (Delta, Jagged) are type I transmembrane glycoproteins that communicate by attaching to membrane-bound ligands on neighboring cells. The majority of reported Notch functions need Regulated Intramembrane Proteolysis (RIP) and belong to the cleavage-dependent or canonical Notch signaling pathway (31).

**Figure 2:**
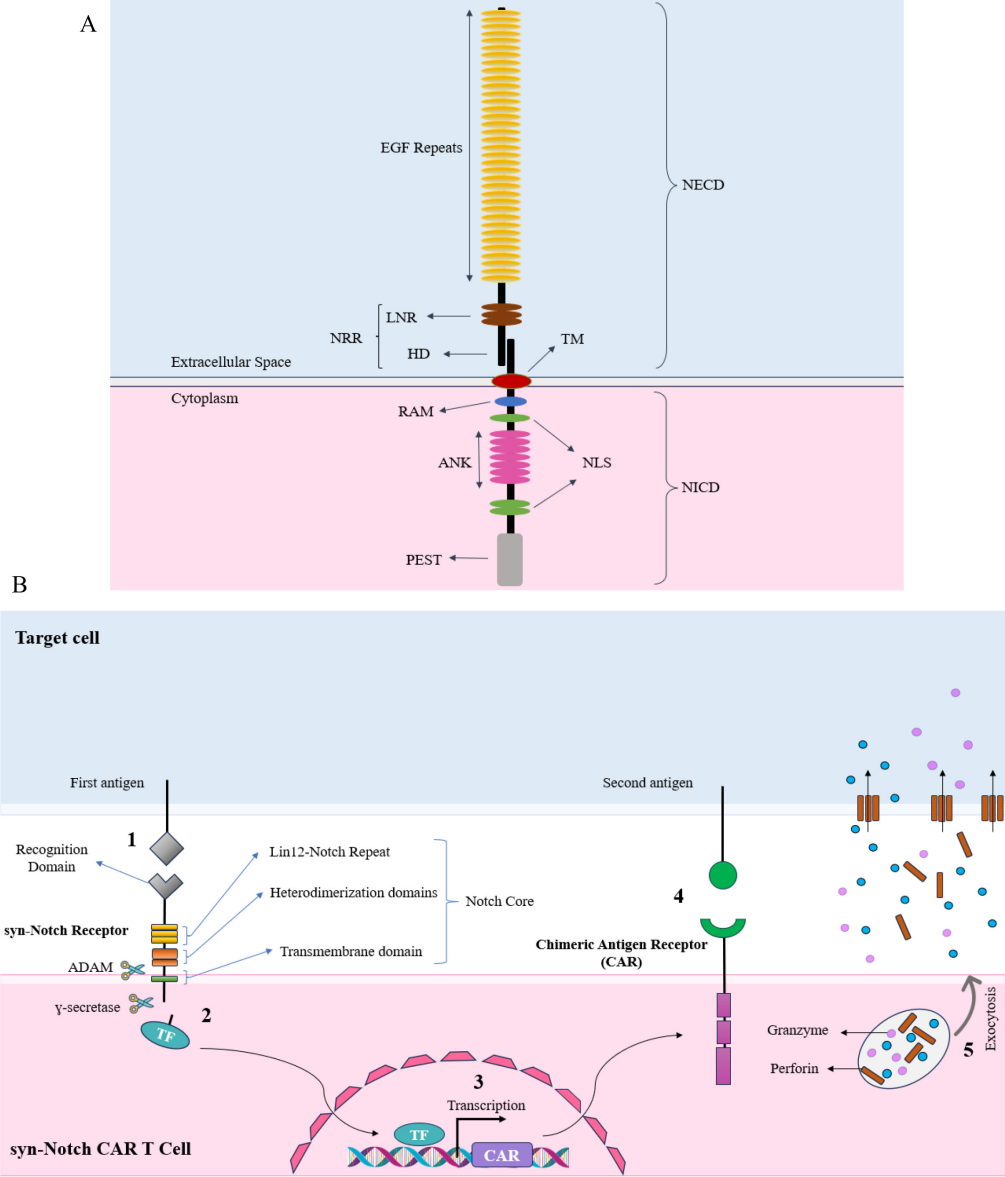
**(A)** Structural components of a canonical Notch receptor. The illustration highlights the major domains of the Notch receptor. The extracellular domain (NECD) consists of multiple EGF repeats, essential for ligand binding, and the Lin12-Notch repeat (LNR) region, which is located close to the transmembrane (TM) domain and cell membrane, which prevents ligand-independent activation. The Notch receptor is kept in an inactive state in the absence of its ligand primarily by its negative regulatory region (NRR). This region—including the LNRs and the heterodimerization (HD) domain—folds in such a way that it shields the critical S2 cleavage site from ADAM metalloproteases. The intracellular domain (NICD) includes the RBPJk-associated molecule (RAM) domain, ankyrin repeats (ANK), nuclear localization signals (NLS), and the PEST sequence. Then the receptor undergoes proteolytic cleavage, which results in the release of the NICD upon ligand binding. The NICD translocates to the nucleus, where the RAM domain and ankyrin repeats (ANK) facilitate interaction with transcriptional co-activators. The PEST sequence regulates the receptor’s degradation, while the nuclear localization signal (NLS) guarantees accurate nuclear localization. Collectively, these components coordinate the activation of target gene transcription, thereby transmitting the extracellular signal to the nucleus. **(B)** An overview of the synNotch CAR-T cell Mechanism of Action. In the synNotch circuit, the detection of the first antigen triggers the release of the transcription factor from the synthetic Notch receptor, leading to the expression of the chimeric antigen receptor (CAR). The CAR, appearing on the T cell membrane, then detects the second antigen on the target cell. Upon detection of the second antigen, the T cell becomes activated and subsequently secretes perforin and granzyme, leading to the death of the target cell.

The Notch-Delta pathway initiates with the synthesis of a monomeric form of the Notch protein within the endoplasmic reticulum. Subsequently, the Notch protein migrates to the Golgi apparatus. Within the Golgi complex, proteolytic cleavage occurs, dividing the Notch protein into two segments. Remarkably, these two parts remain connected through a non-covalent bond. Eventually, the mature Notch protein translocates to the cell membrane surface. The Notch receptor consists of a large extracellular domain (NECD) and a somewhat smaller intracellular domain (NICD). The NECD is composed of up to 36 tandemly arranged epidermal growth factor (EGF)–like repeats, followed by three similarly arranged Lin12-Notch (LN) repeats, which are exclusive to the Notch receptor family. The NICD contains the RBPJk-associated molecule (RAM) region in the juxtamembrane region, followed by seven ankyrin repeats (ANK), a putative transactivating domain (32) and a C-terminal PEST motif. The EGF-like repeats contain the receptor’s ligand-binding sites (33–35), whereas the LN repeats play a role in preventing ligand-independent signaling (36–38). The entire intracellular part of the receptor, the NICD, is involved in relaying signals to the nucleus (39–41). (**Figure 2A**).

Notch receptor activation is mediated by a sequence of proteolytic events. Ligand binding leads to an “ectodomain shedding-like” cleavage event (42, 43). Cleavage occurs at an external site (S2), around 12 amino acids distant from the transmembrane domain, between the Ala1710 and Val1711 residues. The carboxy product of S2 cleavage is referred to as NEXT, which stands for Notch Extracellular Truncation. Metalloproteases are thought to be responsible for cleaving S2. The metalloprotease TACE/ADAM17 was shown to cleave Notch at the S2 site *in vitro* (42).

NEXT is progressively cleaved by γ-secretase within the transmembrane domain, with the cleavage process beginning near the inner plasma membrane leaflet at site 3 (S3) and concluding near the middle of the transmembrane domain at site 4 (S4). The NICD is only able to translocate to the nucleus after γ-secretase cleavage. NICD initially interacts with the DNA-binding protein CSL [CBF1/RBPjκ/Su(H)/Lag-1] through its RAM domain. The coactivator Mastermind/Lag-3 is recruited by the ANK domain of NICD, which then associates with CSL to help recruit the MED8 mediator transcription activation complex. This interaction induces the upregulation of downstream target genes (44). By fusing Gal4 and Gal4-VP16 to the intracellular region of the Notch receptor in previous studies, researchers provided evidence that the Notch intracellular domain (NICD) translocates to the nucleus and influences gene expression there (39, 40).

Gordon et al. subsequently developed two artificial ligand-receptor systems that exhibited the ability of signal-sending cells to increase metalloprotease sensitivity in the NRR (negative regulatory region), which is part of the extracellular region following the EGF repeats, in the absence of native ligand-receptor interactions. This indicates that ligand binding does not need to have an allosteric effect on the sensitivity of the NRR for activating proteolysis to happen. These findings show that the mechanical force sent by signal-sending cells is enough to unfold the NRR and make Notch more vulnerable to being activated by proteolysis (45).

Inspired by the Notch signaling pathway and building on the pioneering work of the Lim Lab, a synthetic Notch protein (synNotch) can be engineered with an extracellular domain that includes a single-chain variable fragment (scFv), heavy-chain-only camelid antibodies (VHH), or another moiety capable of binding to a specific antigen. The intracellular segment of this synNotch protein would then contain a transcription factor designed to enhance the expression of user-specified target genes (e.g., the CAR or TCR gene) (24, 46). SynNotch receptors are a class of artificial receptors made up of chimeric protein domains. These domains include an extracellular domain that binds to antigens, the Notch juxtamembrane and transmembrane domains, and intracellular domains consisting of orthogonal transcription factors.

In the case of synNotch CAR-T cells, a combinatorial activation circuit can be engineered by inserting the CAR gene downstream of a promoter that is responsive to a specific transcription factor. The initial antigen can be detected by this synthetic Notch receptor (No. 1 - **Figure 2B**), and upon activation, the released transcription factor (No. 2) can trigger the CAR expression (No. 3). This CAR can subsequently identify a second antigen (No. 4), which results in the activation and proliferation of T cells and release of perforin and granzyme (No. 5). It is important to emphasize the specific synNotch-CAR designs that have been employed to date, given the article’s emphasis on synNotch CAR-T cell constructs.

It’s important to note that, although Notch activation is well characterized, the processes behind synNotch activation need further research. Notch and synNotch activation vary substantially in spite of their same basic ideas.

A study demonstrated that while endogenous Notch and FKBP-FRB dimerization-based synNotch (ff-synNotch) require ligand intracellular domain (ICD) endocytosis to generate mechanical force for receptor cleavage, antibody-antigen synNotch (aa-synNotch) does not. Instead, aa-synNotch activation relies on receptor-ligand clustering rather than trans-endocytosis (TEC). Notably, aa-synNotch exhibited reverse TEC, in which ligands were internalized by the receiving cell but remained active. Additionally, the discovery of SNIPRs, a synNotch variant lacking the negative regulatory region (NRR), further highlights that synthetic systems can bypass certain regulatory constraints of endogenous Notch, offering insights into alternative activation mechanisms for next-generation synNotch designs (47).

### 2.2 SynNotch transcription factors

Recent advancements in synthetic biology have expanded the possibilities for designing synthetic transcription factors (STFs) with precise control over gene expression. While transcription factors such as Gal4-VP64, Gal4-KRAB, ZFHD1-VP64, and tetR-VP64 (tTA) have been widely used in the development of synNotch constructs, there may be other options to consider. Synthetic transcription factors contain two domains: DNA binding and activation/inhibition of transcription. Although some transcription activator/repressor domains are reusable in a set of artificial transcription factors, it is imperative that the DNA-binding domain must remain unique for each transcription factor. Various DNA-binding domains have been extensively applied in mammalian cells. These approaches have facilitated some advancements in the research; nonetheless, the quantity of genes applicable in mammalian cells remains restricted (48). In addition, the tet protein family has the capacity to be affected by low molecular compounds (49), whereas Gal4 proteins only identify a short sequence of base pairs (50). These properties are inadequate for the construction of more intricate gene circuits.

Alternative approaches, such as using transcription activator-like effectors (TALE) and catalytically inactive enzymes, Clustered Regularly Interspaced Short Palindromic Repeats (CRISPR) associated protein 9 (dCas9) are worth considering due to their ability to provide accurate and scalable DNA-binding domains capable of selectively adhering to specific sequences (51–53).

Nevertheless, the first one exhibits a repetitive pattern that renders it unstable in viral transductions (54), whilst the second one necessitates a gRNA for its functionality, and there are reported constraints regarding RNA generation in mid-size synthetic gene networks (55). In addition, each of these methods needs encoding sequences of considerable size. To create gene networks of intermediate size in mammalian cells, a multitude of DNA binding domains that can overcome these challenges are necessary.

In a study, researchers investigate the feasibility of using inactivated meganucleases as the DNA-binding component of synthetic transcription factors. They have shown that it is possible to create extremely distinct and effective synthetic activators and repressors by employing deactivated meganucleases. These newly developed transcription factors may then be integrated into synthetic signal proteins and gene circuits. Their findings demonstrate the use of inactivated meganucleases as DNA-binding domains for creating a variety of high-quality synthetic transcription factors. These factors are essential for constructing medium-sized gene circuits in mammalian cells (48).

In addition, most synNotch CAR designs have used mouse Notch1 (M1). However, in a recent study, researchers have developed synNotch receptors utilizing other members of the Notch family from diverse species, such as human (H), mouse (M), Drosophila (Fly), and zebrafish (Z). They showed that the Z3 synNotch receptor showed better activation than the mouse Notch1 (M1) receptor but had higher background noise. Adding EGF repeats reduced the background but also decreased activation efficiency. Shortening the EGF repeat (eZ3) improved activation efficiency while maintaining a tolerable background noise. This study opens the door to enhancing synNotch efficacy by modifying individual components, offering the potential for improved activation and reduced background noise in future applications (56).

### 2.3 SynNotch CAR-T cell designs

Given the focus of this article on synNotch CAR-T cell constructs, it is pertinent to highlight specific synNotch-CAR designs that have been utilized to date.

In a study, Roybal et al. demonstrated combinatorial antigen recognition T cell circuits. These circuits employ synNotch receptors for one antigen to produce CARs for another. They created a CD19 synNotch receptor that produces mesothelin-targeting CARs. SynNotch has an extracellular domain that binds CD19, a minimal regulatory region from mouse Notch1 (amino acids Ile1427 to Arg1752), which spans both the juxtamembrane and transmembrane regions and an intracellular domain that triggers gene expression upon antigen contact. They used Gal4-VP64 or TetR-VP64 (tTa) as transcriptional activators in the synNotch design, which are part of the intracellular domain and increase CAR gene expression. Note that these activators work differently: Gal4-VP64 binds to UAS (Upstream Activation Sequence) sequences to activate transcription, while TetR-VP64 binds to tetO sequences and can be regulated by tetracycline. As part of the structure, they also incorporated an N-terminal CD8a signal peptide (MALPVTALLLPLALLLHAARP) to facilitate membrane trafficking. These dual-receptor T cells become armed and activated through an AND-gate mechanism. These T cells, which are selectively activated by combinations of antigens, have a remarkable ability to distinguish between different types of malignancies and effectively eliminate those associated with multiple antigens while leaving single-antigen tumors unaffected (24).

In a separate study, researchers engineered T cells by incorporating synNotch receptors that specifically targeted EpCAM or B7-H3, which are present on ROR1-positive tumor cells but absent on ROR1-positive stromal cells. These synNotch receptors selectively induced the expression of ROR1 CAR within tumors, leading to tumor regression without adverse effects when tumor cells were isolated from normal ROR1-positive cells. This strategy allows for the safe targeting of tumors that are adequately separated from normal cells (57).

Or researchers used GD2 as the gate and B7H3 as the target in the synNotch CAR design. The development of neuroblastoma was effectively inhibited both *in vitro* and in metastatic xenograft murine models, demonstrating great specificity and effectiveness. These enhancements are partially attributed to the improved metabolic fitness of GD2-B7H3 CAR-T cells, as shown by their naïve T-like post-cytotoxicity oxidative metabolism and reduced exhaustion profile (58).

Or a study conducted by Hyrenius-Wittsten et al., ALPPL2 was discovered as a tumor-specific antigen found in several types of solid tumors, such as mesothelioma and ovarian cancer. ALPPL2 may be specifically targeted either alone or in combination with antigens like MCAM, mesothelin, or HER2 by using synNotch CAR circuits. In murine models, synNotch CAR-T cells demonstrated superior tumor suppression in comparison to T cells expressing CARs constitutively, by inhibiting tonic signaling and maintaining a durable, non-exhausted state. This identifies ALPPL2 as a promising candidate for cell treatment in several types of solid tumors and emphasizes the advantages of synNotch CAR-T cells (59).

Researchers also expanded the synNotch CAR design to natural killer (NK) cells to specifically address colorectal cancer (CRC) with HER2 amplification. They modified NK cells by introducing a synthetic Notch receptor that selectively recognizes HER2. When the synNotch receptor binds to HER2, it triggers the activation of a chimeric antigen receptor (CAR) that specifically targets carcinoembryonic antigen (CEA). This dual-targeting technique ensures that the CAR is only produced when both HER2 and CEA are present, thereby increasing specificity and minimizing off-target effects. This method offers a novel, scalable, and safe off-the-shelf cell therapy with the potential to combat HER2-amplified colorectal cancer that is resistant or only partly sensitive to HER2/EGFR blocking (60).

## 3 Advancing synNotch CAR-T cell circuits for enhanced tumor recognition

### 3.1 3-input AND/OR synNotch circuits

CAR-T cells, which are designed to direct their cytotoxic effects towards a specific extracellular antigen, have led to harmful on-target off-tumor interactions with normal tissues during clinical trials (61–66). Many potential CAR tumor antigen targets in solid cancers are also present in normal epithelial tissues. Bioinformatics studies, on the other hand, show that identifying combinatorial antigen patterns might make it easier to target cancer cells more precisely (67–69). SynNotch-CAR circuits operate as Boolean AND gates, necessitating the recognition of both priming (synNotch) and killing (CAR) antigens. These circuits can also be viewed as “IF-THEN” circuits, as they only carry out CAR-directed killing if they are first primed by the synNotch ligand (70). Consequently, they can potentially present a viable solution to address the previously mentioned challenges.

**Figure 3:**
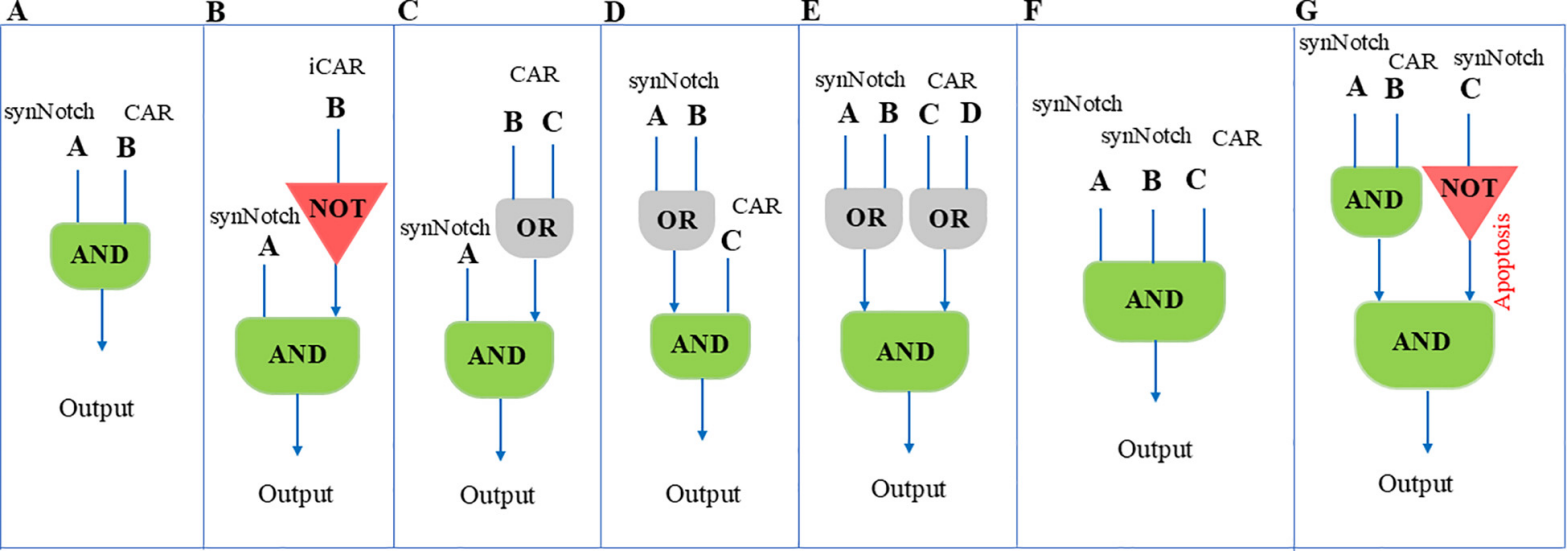
Illustrates different logic gated configurations and their respective behaviors in the context of synNoch CAR-T cell activation. The logical gate designs shown in this figure provide valuable insights into how these systems can be applied to refine antigen targeting and improve therapeutic specificity. **(A)** An AND gate integrating inputs A and B, resulting in T cell activation only when both antigens A and B are concurrently detected. This ensures that CAR-T cells respond specifically to cells expressing both antigens. **(B)** An AND gate combined with a NOT gate, where T cell activation occurs if antigen A is present and antigen B is absent. This enhances specificity by excluding cells expressing antigen B. **(C)** AND-OR gate: Activation is triggered by antigen A along with either B or C. **(D)** OR-AND gate: T cell activation occurs when antigen C is present with either A or B. **(E)** OR-AND-OR gate: Activation is triggered when either antigen A or B is present in combination with either antigen C or D. **(F)** Multi-input AND gate: T cell activation occurs only when all three antigens A, B, and C are present, ensuring high specificity for cells expressing this unique combination of antigens. **(G)** AND-NOT gate: Activation occurs with antigens A and B present, but antigen C absent, adding an exclusion mechanism to refine the targeting. This figure was designed based on inspiration from the article published by Williams et al., Science 370, 1099–1104 (2020).

In a study, researchers created a three-input AND gate using Gal4- and LexA-based synNotch platforms to detect the first and second priming antigens and a CAR to detect the third antigen (**Figure 3F**). These receptor components can be configured like electronic circuits: in series (synNotch A induces synNotch B, which then induces CAR C) or in parallel (synNotch A induces part 1, and synNotch B induces part 2 of a split CAR). They engineered both configurations using synNotch receptors for EGFR and MET. In the series circuit, the dual synNotch cascade induced an anti-HER2 CAR. In the parallel circuit, the two synNotch receptors induced different parts of a split anti-HER2 CAR. The series circuit showed precise recognition in various *in vitro* assays, including cell killing and proliferation, only when target cells expressed all three antigens. In contrast, the parallel circuit was less effective, showing partial killing of one antigen combination (EGFR+/HER2+) (71).

Multi-input circuits are an alternative method to enhance the specificity of T cells against tumor cells. However, tumor cells exhibit intrinsic heterogeneity, resulting in varying surface antigen profiles. Consequently, the activation of multiantigen circuits requires recognition of all relevant antigens. Unfortunately, not all tumor cells express different antigens simultaneously, leading to potential tumor escape. A potential solution to this challenge is to design circuits with recognition flexibility. For instance, the concept of first OR-gated CAR-T cells relies on tandem CARs (72–76). Recognition of either of the two target antigens triggers T cell activation. While this approach inherits some limitations from conventional CAR-T cells, the use of synNotch CAR-T cells—where synNotch receptor activation induces tandem CAR expression—may help overcome some limitations (**Figure 3C**). The application of this strategy can be observed in glioblastoma tumor cells expressing a neo-antigen known as epidermal growth factor receptor splice variant III (EGFRvIII). EGFRvIII is highly specific to glioblastoma and represents a promising target. However, the heterogeneous expression of this antigen on tumor cells can lead to tumor escape (77–83). Conversely, a large number of glioblastoma tumor cells express ephrin type A receptor 2 (EphA2) and interleukin 13 receptor α2 (IL13Rα2). Unfortunately, these antigens are also expressed in normal tissue (84–86). We can overcome these types of limitations in tumors like glioblastoma by designing an ‘IF-THEN’ circuit. This circuit is primed by heterogeneous glioblastoma neo-antigens (like EGFRvIII) and subsequently eliminates target cells using tandem CARs that recognize homogenous but nonspecific antigens in glioblastoma (like EphA2 and IL13Rα2). In cases of EGFRvIII-negative glioblastoma, central nervous system (CNS)-specific antigens (like myelin oligodendrocyte glycoprotein or MOG) can be used to prime T cells (70). The spatially limited expression of tandem CARs near priming cells ensures that normal cells lacking the priming antigen are not targeted. Circuits that are primed by cancer-specific but heterogeneous antigens and then kill cells expressing homogeneous and imperfectly tumor-specific antigens are called trans-killing circuits. This means that the priming and killing antigens are on different but nearby cells. In contrast, we have cis-killing circuits in which priming and killing are based on antigens on the same cells. Furthermore, one solution to precisely locate the killing potency of T cells in the tumor microenvironment and reduce bystander normal cell targeting is to build trans-killing circuits. Additionally, researchers have built tandem synNotch receptors that can recognize EGFR or HER2 (OR-gate) as a priming signal and subsequently induce anti-MET CAR expression (**Figure 3D**). The results show that these cells can have promising results in detecting the target cells based on a single positive priming antigen (71).

In 3-input circuits, the effectiveness of the first and second transcription factors released from synNotch receptors, along with the antigen density on target cells, plays a critical role in initiating signaling cascades and generating detectable killing outputs. Experiments to find suitable transcription factors in series configuration show that if the first transcription factor has a strong capability to induce the expression of downstream genes (like Gal4) and the second transcription factor is slightly weaker (like LEXA), these 3-input AND-gate circuits can yield promising results. Considering that CARs are more sensitive than synNotch receptors, it is advisable to use the antigen with the lowest expression level as the CAR ligand (71). All these approaches help scientists build circuits that work on a sequential recognition process and can detect targets precisely.

### 3.2 OFF-notch receptor

While most types of cancer don’t have unique surface markers, the fact that target antigens can be found in healthy tissues has led to major on-target off-tumor side effects in new CAR-T cell therapies (61, 63, 87–89). To address the issue of on-target off-tumor side effects, one potential approach is to combine a cancer antigen with a non-cancer antigen in a NOT-gate manner. While the cancer antigen is present on both cancerous and healthy cells, the non-cancer antigen is unique to healthy cells. When the non-cancer antigen is present, it blocks CAR-T cell activation against the cell expressing it (90, 91).

The concept was initially introduced with inhibitory chimeric antigen receptors (iCARs), which feature an antigen recognition domain linked to a cytotoxic T lymphocyte-associated protein 4 (CTLA4) or a programmed cell death protein 1 (PD-1) signaling domain. This fusion activates an antigen-specific immune checkpoint when a non-cancer antigen is present (90). Others have adapted this strategy by identifying alternative inhibitory signaling domains from receptors such as TIGIT, LILRB1, and BTLA as suitable for iCAR design (92–95). Combining iCARs with AND-gated CARs can enhance safety by improving specificity and reducing off-target effects. This integration of positive and negative regulation allows for more precise and effective T cell responses. However, iCARs have limitations, such as both CAR structures should be expressed in equal and high quantities. Additionally, the selected antigen must have a balanced affinity to the CARs. Moreover, the density of the target antigen plays a crucial role in iCAR function, as insufficient or unequal expression can impair the inhibitory mechanism. These limitations hinder the robust use of iCARs.

In a study conducted by Williams et al., the researchers constructed a synNotch circuit that activates the expression of the proapoptotic factor tBID, referred to as OFF-Notch, which induces rapid T cell death upon stimulation. To incorporate this OFF-Notch receptor as a NOT gate in a three-antigen circuit that also includes an AND gate, they used two orthogonal synNotch platforms with distinct transcriptional regulatory domains (GAL4-VP64 and LEXA-VP64). They combined an anti-HER2 NOT gate with an AND gate recognizing GFP and CD19. The composite circuit demonstrated selective targeting, sparing tumor cells expressing HER2 (NOT antigen) and killing only those expressing GFP, CD19, and NOT HER2 (**Figure 3G**). This selectivity was also observed in T cell proliferation assays. The study highlights the importance of matching kinetics for circuits incorporating both positive and negative regulation, as the NOT gate worked best when counteracting a synNotch-induced CAR rather than a constitutively expressed one (71). Hence, it’s essential to design a multiantigen circuit that can activate and compete on a similar timescale and kinetically match signaling pathways. Moreover, analyzing the “surfaceome” of both normal and malignant cells helps us to identify the optimal antigens (96). This will ensure the effectiveness of the approach.

**Figures 3A–G** shows various logic-gating techniques that can be used with synNotch CAR-T cell structures. In order to prevent tumor immune evasion—in which cancer cells may downregulate or alter individual antigens—and to lower the risk of off-tumor toxicity by requiring more rigorous, multi-antigen recognition, these modular frameworks demonstrate how T cells can be precisely programmed to trigger activation only under specific antigenic conditions.

### 3.3 Targeting intracellular antigens

Endeavors to enhance and broaden the application of synNotch receptors have led to remarkable advancements and novel innovations in this field. Scientists are attempting to construct more intricate logical circuits to increase precision and limit the activation of T cells in precise locations, surpassing the capabilities of the first-generation AND-gate circuit.

Most traditional CARs guide T-cells towards external antigens, but their broad use has been limited due to the scarcity of tumor surface antigens (97, 98). It’s known that more than 75% of cell proteins are intracellular, many of which are cancer antigens (99). Many tumor antigens that control cellular metabolism are intracellular molecules, which conventional strategies cannot detect (100–102). Intracellular antigens can be naturally processed into short peptides, which are then presented on the cell surface by major histocompatibility complex (MHC) molecules as MHC/peptide complexes (100, 103). Therefore, if we design a recognition circuit against MHC and the proteins in the MHC cleft, we can detect numerous intracellular cancer antigens and distinguish between normal and cancer cells.

In a study, researchers aimed to determine if nanobody (Nb)-based T cell receptor (TCR)-like CAR-T cells could target and kill tumor cells by focusing on MHC/peptide complexes. They selected Glypican-3 (GPC3) and Wilms tumor 1 (WT1) oncoproteins as examples. Using the immune nanobody phage display library, they developed HLA-A2/GPC3- and HLA-A2/WT1-specific nanobodies and incorporated them into TCR-like CARs. These TCR-like Nb CAR-T cells selectively recognized and destroyed MHC/peptide complex-expressing tumor cells in both *in vitro* assays and subcutaneous mouse tumor models (104).

In another study, researchers identified mAb 2D2 as a novel TCR-like human full-length IgG1 monoclonal antibody targeting the intracellular cancer-testis antigen NY-ESO-1 presented by HLA-A2 molecules. The mAb 2D2 specifically recognized HLA-A2/NY-ESO-1 complexes and demonstrated the ability to lyse tumor cells both *in vitro* and *in vivo* when engineered into 2D2-CAR-T cells. Mice treated with 2D2-CAR-T cells showed significant tumor growth inhibition and enhanced survival without adverse effects, highlighting the safety and efficacy of this approach. Previous studies on TCR-like antibodies showed that increasing antibody affinity could lead to cross-reactivity with HLA molecules. However, mAb 2D2 maintained a moderate affinity, promoting CAR-T therapy success without off-target issues. The study also compared the efficacy of 2D2-CAR-T cells with A2-ESO TCR-T cells, finding similar antitumor responses but lower cytokine production in 2D2-CAR-T cells, reducing potential toxicity. Further research is needed to improve 2D2-CAR-T cell trafficking and persistence in solid cancers and to explore combination therapies for enhanced antitumor activity (105).

Additionally, in a study, researchers designed a recognition circuit to identify melanoma cells by targeting both MET and MART1 antigens. They developed an anti-METsynNotch→anti-MART1 TCR circuit, which selectively targeted MET+/MART1+ melanoma cells without affecting normal melanocytes. This approach improved the specificity of therapeutic TCRs. Additionally, they engineered CARs using scFvs against specific peptide-MHC complexes, validating an in Notch receptor with an HLA-A2/AFP recognition domain. T cells with this receptor acted as AND gates, specifically killing dual-positive target cells (71).

Together, these findings highlight the potential of utilizing TCR-like antibodies in synNotch CAR-T cell therapies to further revolutionize cancer treatment by offering more targeted and effective therapeutic options.

### 3.4 Targeting *de novo* antigens

Identifying target antigens to build combinatorial recognition platforms poses challenges, including heterogeneity, instability, and off-tumor expression of antigens, alongside fluctuating expression patterns observed among various patients and tumors. By creating new targets for CAR or synNotch receptors in cells that we aim to eliminate, we can overcome some major limitations. This technique is based on expressing new and unique synthetic antigens on target cells.

In the initial reports on this approach, a group attempted to express murine CD19 (mCD19) on cancer cells (both *in vitro* and *in vivo*) using the vaccinia virus and treated them with CD19 CAR-T cells (106).

Park et al. also attempted to express a truncated form of CD19 (tCD19) on different tumor cells using an oncolytic vaccinia virus coding for CD19t (OV19t) and co-cultured CAR-T cells with infected cells. Furthermore, to expand this method, they tested it in mouse models. By administering a combination of the oncolytic virus and CAR-T cells against tCD19, they demonstrated minimal off-target effects. They also tested the feasibility of this approach by delivering non-human specific antigens, but more research is needed in this area to address clinical limitations (107).

One of the advantages of using oncolytic viruses to deliver neo antigens to tumor cells is that these viruses can also have a synergistic effect. They can kill target cells through the lytic cycle and enhance antigen spreading in the TME by lysing tumor cells, which can enhance the potency of CAR-T cells, as proven by previous studies (107–110).

Additionally, oncolytic viruses may make solid tumors more accessible, and CAR-T cells may be able to penetrate better because they can convert cold tumors into hot tumors (111, 112).

To date, only one oncolytic virus therapy (HSV-1-based TVEC) has received FDA approval. The majority of clinical studies on oncolytic viruses have focused on local administration to the cancerous tissue. Therefore, the systemic administration of these therapies presents a current challenge that requires further research. Another consideration is that while oncolytic viruses have an intrinsic tendency to infect tumor cells, they may also infect some normal cells, which could cause severe side effects in combination therapy with CAR-T cells.

Another study leverages the benefits of amphiphilic polyethylene glycol (PEG)-lipids to decorate cells both *in vitro* and *in vivo*, using an amph-ligand structure to redirect CAR-T cells. They selected fluorescein isothiocyanate (FITC) as a non-immunogenic and safe ligand for CAR-T cells. By injecting amph-FITC intratumorally and administering CAR-T cells systemically against FITC, they reported that CAR-T cells could be activated in solid tumors (113).

VHHs are a versatile and useful tool in immunotherapy because they can be used in many ways. For example, they can be used as effective neutralizing agents or as recognition moieties in CAR constructs (114, 115).The use of adeno-associated virus (AAV) or mRNA to deliver the antigen-binding fragment of the VHH as a neo-antigen to various tumor types has also been reported. Given that VHH is thermally stable and resistant to proteases and extreme pH conditions, it appears to be a promising candidate for a neo-antigen (116).

Recently, Vincent et al. utilized tumor-colonizing probiotic bacteria (*Escherichia coli* Nissle 1917) that can release a new antigen in TME to guide CAR-T cells. They tagged tumor cells with superfolder green fluorescent protein (sfGFP) and evaluated the killing potency of probiotic-guided anti-sfGFP CAR-T cells. This platform can also induce inflammatory reactions that may enhance antitumor responses. However, the potential toxicity of systemic administration of bacteria may raise concerns about its use in clinical investigations (117).

These platforms can address some major challenges in treating solid tumors, such as antigen bottleneck and difficulty in inserting multitransgen in logic-gated circuits.

In the process of choosing optimal neoantigen candidates for expression on tumor cells, several factors need consideration. These include the antigen’s size, its suitability for genetic encoding and delivery, detectability via scFv or VHH, stability in expression on tumor cells, and the natural immune response elicited by the antigen.

By employing this technique, we have the potential to introduce a wide range of antigens as new targets for synNotch and CAR-T cells. This approach appears to be a fundamental solution to overcoming the limitations of antigen selection in the current platforms.

## 4 SynNotch receptors for targeted cytokine secretion

The use of CAR-T cells expressing specific cytokines to enhance anti-tumor activity is like an old wine in a new bottle. While this strategy has demonstrated some benefits, it also presents limitations, such as systemic toxicity and sensitivity to tumor suppression.

Previous research has already demonstrated the effectiveness of synNotch T cells secreting CXCL10. In a humanized mouse model, these synNotch T cells significantly inhibited tumor growth, increased CD3+ T cell infiltration, and elevated CXCL10 and IFN-gamma levels at the tumor site. Minimal increases in CXCL10 and IFN-gamma in the serum suggest that the treatment is safe and holds potential for clinical applications (118).

Then in research conducted by Allen et al., researchers have constructed a circuit incorporating a synNotch receptor, which induces the expression of an IL-2 transgene, alongside a CAR construct targeting a specific antigen. Their findings suggest that this circuit could serve as an alternative to CAR-T cells that constitutively express IL-2, CAR-induced IL-2 expression, or systemic administration of IL-2. Moreover, synNotch-induced IL-2 expression has the potential to overcome TME suppression and drive T cells to immune-excluded tumors (119).

This platform can also be applied in various other fields. For example, one of the main obstacles in the fields of autoimmune and inflammatory disorders and tissue transplantation is the fact that immunosuppressive medications used to treat these conditions frequently cause systemic effects and other negative repercussions. Reddy et al. have used synNotch technology to create synthetic suppressor cells that create an immunosuppressive milieu locally to solve this issue. This group’s most effective strategy uses cells that imitate important developmental cues of regulatory T lymphocytes (Tregs) by acting as suppliers of anti-inflammatory cytokines and sinks of pro-inflammatory cytokines.

This platform’s mechanism works as follows: A synNotch construct, which is incorporated into CD4 T cells, recognizes the target antigen and initiates the downstream production of two essential components. The first is TGF-β, a suppressive cytokine that helps establish an immunosuppressive milieu. The second is the cytokine sink CD25, a subunit of the high-affinity IL-2 receptor complex. CD25 has two roles on this platform: A) IL-2 depletion limits the effector activity of conventional and CAR-T cells by inhibiting their activation and proliferation through IL-2 sequestration. B) Autocrine expansion of synthetic suppressor cells: CD25 stimulates the growth of these designed suppressor cells by ensnaring IL-2, enabling them to take control of the microenvironment.

This approach has demonstrated the protection of specific tissues from off-target CAR T-cell toxicity without compromising the effectiveness of tumor eradication. Additionally, in a pancreatic islet transplantation model, this system successfully protected the graft from cytotoxic T-cell attack, highlighting its potential in transplant immunology. This synthetic suppressor cell system can be applied in scenarios where on-target, off-tumor toxicity needs to be mitigated in cell-based cancer therapies. It also holds promise for treating autoimmune diseases and preventing graft rejection in tissue transplantation (120).

One of the conundrums in the field of targeted drug delivery—particularly to anatomically restricted tissues such as the central nervous system (CNS)—can be addressed using the synNotch platform. This system enables precise targeting and localization of therapeutic agents within hard-to-reach tissues. Combining this platform with the intrinsic ability of T lymphocytes to traverse the blood-brain barrier (BBB) introduces the concept of tissue-sensing T cells.

In this approach, anatomical cues are utilized to guide T lymphocytes toward the target tissue, where they subsequently execute their specialized function—either expressing a therapeutic construct against a disease-specific antigen or producing immunomodulatory cytokines.

Simic et al. have developed this platform for CNS-related diseases, including brain tumors, brain metastases, neuroinflammation, and neurodegenerative disorders. They identified brevican (BCAN), an extracellular matrix antigen in the CNS, as a synNotch target. Consequently, the engineered cells initially localize within CNS-associated tissues, where they exert their therapeutic effects in a disease-specific manner. Depending on the pathological condition, these cells can either secrete interleukin-10 (IL-10) or express a CAR receptor against a disease-associated target, ensuring localized immune modulation (121).

This conceptual framework is highly adaptable and can be generalized to other tissues, expanding the potential applications of synNotch-based precision immunotherapy across various disease contexts. Thus, the integration of synNotch technology into CAR T-cell therapy and other immune-based treatments holds great promise not only in cancer immunotherapy but also in treating autoimmune diseases, tissue transplantation, and even CNS disorders. The flexibility and precision of the platform make it a powerful tool for overcoming existing challenges and paving the way for targeted, localized therapies in a range of clinical applications.

## 5 Utilizing adaptors in synNotch CARs

CAR design has witnessed continuous alterations from the first to the fourth generation, as previously mentioned, which have enhanced the proliferation, cytotoxicity, secretion of cytokines, and *in vivo* persistence of CAR-T cells (122). Adaptor CAR-T cells were developed as well to enhance conventional CAR-T cell treatment. Adaptor CARs have the same fundamental structure as conventional CARs, except that the extracellular domain interacts with a binding partner integrated into the adaptor molecule rather than a tumor-associated antigen. The bifunctional adaptor molecule, in turn, offers tumor selectivity while also acting as a linker at the tumor-adaptor CAR-T cell interface. As with ordinary CAR-T cells, this combination may then trigger anti-tumor responses (123). The adapter molecule is essential for CAR T cytotoxicity, which allows for the temporal regulation of T cell activation, expansion, and persistence (124).

In this section, we will not discuss the design strategies for creating an adaptor CAR T. For additional information, please refer to the article by Amelia C. McCue et al.,”Advances in Modular Control of CAR-T Therapy with Adapter-Mediated CARs,” Advanced Drug Delivery Reviews (124).

Building upon the foundation of adaptor CAR-T cells, SNAP-CAR and SNAP-synNotch receptors have emerged as innovative strategies to further enhance modular and adaptable immunotherapies. These systems utilize universal recognition circuits, where the targeting specificity can be controlled post-production through the covalent attachment of co-administered antibodies with a benzylguanine (BG) motif. In these receptors, a SNAPtag self-labeling enzyme is fused to the receptor, reacting with BG-conjugated antibodies to program antigen recognition. They demonstrated that SNAP-CAR and SNAP-synNotch receptors can be effectively targeted by clinically relevant BG-conjugated antibodies, showing anti-tumor activity in a human tumor xenograft mouse model. Additionally, they developed a mathematical model to better understand the parameters affecting universal receptor signaling. SNAP receptors offer a powerful method to reprogram the targeting specificity of engineered cells post-translationally (125).

To improve the safety and manageability of the synNotch platform, it could incorporate an on/off switch by expressing an apoptotic factor. These enhancements would assist us in controlling its function, particularly in instances where normal cell antigens are recognized.

## 6 Affinity and density: dark side of synNotch CAR-T cell therapy

One aspect of synNotch CAR-T cell research that requires intensive investigation is the affinity of the antigen-binding domain (ABD) within the CAR structure, as well as the antigen density on tumor cells.

It is well known that cancer cells often express surface antigens in higher quantities compared to normal cells. We can leverage this characteristic of cancer cells to enhance the efficacy and specificity of T-cell therapy in solid tumors. Observations have shown that T-cell activation depends on the strength of TCR interaction (126, 127). This observation leads to questions about whether synNotch or CAR affinity could play a role in cell discrimination.

In this context, the effects of two CAR-T cells with differing affinities against EGFR have been assessed. One of the T cells contains a nimotuzumab-CAR, which exhibits low affinity to EGFR, while the other carries a high-affinity cetuximab-CAR. This article ultimately suggests that tuning the sensitivity of CAR-T cells to the antigen density of cancer cells equips the T cells with an approximate ability to distinguish between normal and cancerous cells, all while preserving anti-tumor activity (128).

In a separate study, the ABD affinity of CAR in solid tumors was examined, and it was found that ABD with moderate affinity yielded promising clinical results (129).

To evaluate this proof-of-concept in the synNotch strategy, density-dependent recognition circuits capable of distinguishing the antigen density of target cells have been constructed. This circuit includes a synNotch receptor with a low-affinity scFv against HER2. Upon activation of this receptor, a CAR construct containing a high-affinity scFv against HER2 is expressed. Testing this circuit both *in vitro* and *in vivo* suggests that synNotch CAR-T cells can differentiate between normal cells with weak HER2 expression and cancer cells with higher HER2 expression. Furthermore, T cell activation and CAR expression are limited to tumor cells with HER2 expression increased by 100-fold (130). This behavior, where a minor change in the input signal can trigger sigmoidal changes in the output response, is referred to as ultrasensitive responses (131, 132). Ultrasensitive circuits could potentially help overcome limitations in solid tumor treatments, enhance the ability to discriminate between tumor and normal cells, and also reduce off-target effects.

If future research can establish a mathematical model for the affinity threshold of synthetic receptors based on the antigen density on target cell surfaces, and if we can better control the expression levels of synthetic receptors on immune cells, the current platforms could see significant functional expansion in clinical applications.

## 7 The Yin and Yang of synNotch receptors: unraveling the positive and negative aspects and their boundaries

Upon utilizing synNotch CAR-T cells, the potential for T cells primed by single antigen-expressing cells to migrate and target bystander single-positive cells was a safety consideration that needed to be addressed. However, no strong evidence was found for the migration of primed AND-gate T cells. In In a study investigating this issue, the durability of a CAR construct labeled with green fluorescence upon activation via a synNotch receptor was assessed. After the synNotch engagement ceases, the CAR’s half-life was tested and found to be approximately 10 hours. Because of its short half-life, it might not be able to sustain a strong cytotoxic or proliferative response in tissues that only express one antigen, which would limit its extended activity outside of the priming milieu (70). Additionally, strong local activation of T cells at dual antigen sites, including induced CAR expression and IL-2 release, results in a positive feedback loop that increases T cell activity within the tumor. In conclusion, the combinatorial circuit effectively distinguishes between target tumor cells and normal cells (24).

Another important consideration for using synNotch receptors in clinical applications is their potential for ligand-independent activation. This type of activation is common in canonical Notch receptors, as it has been shown to be essential for the normal development of Drosophila blood cells (133). However, this characteristic may pose a challenge in clinical settings, where unintended activation of the receptor could occur. While the rational for overcoming this issue is to choose cells that express synNotch and only activate in the presence of the antigen on sender cells. This clonal selection procedure is time-consuming and limits the broader deployment of synNotch (134).

Altering the receptor may mitigate these unintended activations; however, the feasibility of achieving clinical applicability remains uncertain. In a study, researchers demonstrated that incorporating an intracellular hydrophobic sequence (QHGQLWF, referred to as RAM7) from native Notch significantly reduces ligand-independent activation. Their enhanced synthetic Notch receptor (esNotch) resulted in a remarkable reduction in ligand-independent activation while preserving the receptor’s capacity for antigen-induced activation. However, cells expressing the esNotch displayed a diminished overall response intensity as a consequence of this modification (134).

Another limitation that synNotch CARs face is their potential for immune rejection due to non-human components, the absence of clear design guidelines for adjustable activity, and their large and complex structure. Efforts to create a human version based on mouse Notch1 resulted in poor performance and unwanted signaling, with both human and mouse versions being incompatible with various transcription factors. To address these issues, researchers systematically developed SNIPR (Synthetic Intramembrane Proteolysis Receptors) so researchers developed synthetic receptors called SNIPRs, similar to Notch receptors. They found that optimizing the extracellular (ECD), transmembrane (TMD), and juxtamembrane (JMD) domains is crucial for performance. By avoiding exposed protease sites and minimizing ECD length, they improved specificity. TMDs and JMDs can be fine-tuned through mutations for clinical needs. Their systematic exploration identified well-expressed receptors that activate reliably, important for cell therapy. SNIPRs rely on ADAM protease and γ-secretase activity, with enhanced signaling during T cell activation. Customizable SNIPRs offer spatial discrimination, sensitivity to various antigen levels, and compatibility with human components, benefiting immunotherapies like CAR-T cells by providing controlled therapeutic responses and reducing issues like T cell exhaustion and systemic toxicity (135).

Numerous strategies have been explored to mitigate the immunogenicity of both CAR‐T and synNotch receptors due to their non‐human components. One widely used approach to reduce the immunogenicity of the antigen‐recognition domain is to “humanize” the scFv or VHH regions. Techniques such as complementarity-determining region (CDR) grafting (136), framework shuffling (137), and site-directed mutagenesis (138) are commonly employed to maintain antigen-binding affinity while minimizing foreign epitopes. Some computational approaches can also be applied to humanize scFv and nanobodies. In a recent study, we employed in silico techniques to humanize an anti-CD19 nanobody and assessed its impact on several functional aspects of CAR-T cell activity (139). Alternatively, scFvs can be sourced from non-immune human antibody libraries to inherently lower immunogenicity. In our laboratory, we have recently isolated a scFv against the CD20 antigen, and we are currently developing CAR‐T and synNotch CAR‐T cells based on this human-derived scFv (140). However, antibodies derived from non-immunized human libraries typically exhibit low affinity and often require affinity maturation through protein engineering techniques to enhance their binding properties (141). It is also important to note that high affinity may result in additional adverse effects, therefore it is not always suitable in the context of CAR-T cell therapy (129).

A potential hypothesis is that even if all individual components of CAR and synNotch receptors are humanized, their novel, non-natural assembly might lead to the formation of unique conformational epitopes. Consequently, these engineered receptors could still be immunogenic. However, to our knowledge, this possibility has not yet been reported in the literature.

Limitations such as balancing CAR expression, using CARs with similar affinity to both antigens and matching the signaling kinetics of both CARs, which previously hindered the application of conventional AND-gated CAR-T cells (for example, tandem CAR-T cells), are almost addressed in synNotch circuits. Moreover, the separation of the downstream signaling pathways of the synNotch receptor and CAR makes it a safer strategy, eliminating concerns about signaling interference.

Existing challenges, such as the selection of ideal antigens, the density of target antigens on tumor cells, fluctuating patterns of antigen expression across different patients and tumor cells, and numerous obstacles in treating solid tumors (like inhibitory conditions in the TME and physical constraints that limit the trafficking and motility of T cells), require researchers to propose potential solutions. The synNotch platform has the ability to be designed to recognize more than three antigens, which can enhance its specificity. However, multi-recognition circuits could lead to insufficient tumor cell killing due to the heterogeneous expression of antigens. Furthermore, in multi-recognition T cells, scientists need to insert multiple transgenes, which makes it challenging and less efficient to construct circuits with more than three input logic gates.

Prior to the synNotch strategy, the concept of having T cells that are fully inhibited in the absence of target antigens and fully activated when both antigens are present was a challenging goal. However, it now appears that we have greater control over T cell activation and proliferation. While synNotch, particularly in CAR-T cells, requires further development to address existing challenges, it remains a promising tool with significant potential for future medical applications. **Table 1** offers a comparative summary of various logic-gate strategies, including IF/THEN-GATE synNotch CAR-T cells.

**Table 1:** Comparison of different logic-gated circuits based on their mechanism of action, CAR expression, specificity, safety, effectiveness, and persistence.

Logic Gate circuits	IF/THEN-GATE (SynNotch CAR-T cell)	AND-GATE CAR-T cell	OR-GATE CAR-T cell
Features			
Mechanism of action	Sequential antigens detection	Simultaneous recognition of multiple antigens	Activates eighter by any target antigens
CAR expression	Inducible (Spatiotemporally controlled)	Constitutive	Constitutive
Specificity	High specificity by Sequential activation	High specificity but depends on co-expression of both antigens	Lower specificity, as it can activate by any of target antigens
Safety	Very high - Activity is strictly confined to tumor sites, reducing on-target/off-tumor toxicity	High - Reduces off-tumor effects	Moderate to Low - May target normal tissues expressing any of the recognized antigens
Effectiveness against tumor heterogeneity	High - Capable of killing adjacent tumor cells with different antigen profile (Trans Killing)	Low to moderate - Risk of tumor escape by lack of co-expression or one antigen loss	High - Covers diverse antigen profiles
Exhaustion and persistence	Lower exhaustion with temporary CAR expression and reduced tonic signaling, ensuring better persistence	Moderate exhaustion, as continuous CAR expression	Higher exhaustion due to persistent activation by multiple antigens

•spatiotemporally controlled: Refers to gene expression that is activated only under specific conditions, controlled in both time (temporal) and location (spatial) to limit off-target effects.

•Off-tumor effects: Unintended targeting and destruction of healthy tissues that express the same antigens as tumor cells, leading to toxicity.

•Trans killing: The ability of CAR-T cells to kill tumor cells that do not express the target antigen themselves but are adjacent to antigen-positive cells.

Logic Gate circuits features

•Tumor heterogeneity: The presence of diverse cell populations within a tumor, with varying expression of antigens, posing a challenge for targeted therapies.

•Exhaustion: A dysfunctional state of T cells characterized by reduced proliferation, cytokine production, and cytotoxic activity due to prolonged stimulation.

•Tonic signaling: Antigen-independent, continuous signaling from CARs and synNotch even in the absence of their target, which can lead to premature T cell exhaustion and reduced therapeutic efficacy.

## 8 Discussion

In recent decades, there has been a significant global effort among scientists to find treatments for various types of cancer. This surge is due to the increasing number of people diagnosed with cancer and the consequent rise in cancer-related mortality. Unhealthy lifestyles and the prevalence of carcinogens in our environment suggest that we can expect even higher statistics in the coming years.

Numerous drugs and chemical agents have been introduced to destroy cancer cells, but the biggest challenge in cancer treatment is the targeted delivery of these therapeutic agents to cancer cells. In the body of a patient, only cancer cells should be exposed to our therapeutic agent, and no harm should come to healthy cells. For instance, in the context of CAR-T cell therapy, the lack of antigens that are exclusively expressed on cancer cells has resulted in numerous side effects (on-target off-tumor) after injecting CAR-T cells into patients. Therefore, it seems that in the future, instead of looking for new therapeutic agents, we should find solutions for targeted delivery and enhance specific targeting of cancer cells by inspiring cell-cell interaction mechanisms.

Since its introduction in 2016, synNotch technology remains relatively new and has many unanswered questions. As demonstrated by the fact that the majority of research is still being done at the animal level and that there is now only one registered clinical trial (NCT06186401), its clinical usefulness remains limited.

Furthermore, the constraints of delivery methods and systems make it difficult to transfer many synNotch structures and CAR constructions in logic gate platforms into cells. It is necessary to find ways to reduce the size of structures and improve transmission techniques. The structures’ immunogenicity and the ligand-independent signaling present additional difficulties that must be resolved. Non-human orthogonal transcription factors that could be immunogenic, and alternative transcription factors or structure-guided deimmunization may be necessary for clinical application.

Another major challenge is the large-scale production of lentiviral or retroviral particles. These challenges encompass factors such as the complexity of production, low yields, scalability issues, purification difficulties, long-term storage concerns, and regulatory compliance. Additionally, the ultimate cost of the product continues to be a significant limiting issue in this industry, based on data from the industrialization of CAR T-cell products. As a result, there is still a long way to go before this technology can be applied extensively in clinical settings. It may be necessary to conduct additional research in order to address this problem by altering the amino acid sequences of the receptors and their domains.

Antigen selection is another main restriction in synNotch CAR T-cell treatment, especially for solid tumors. There are potential situations where synNotch receptors may fail to distinguish tumor and normal tissue. For example, extensive tumor metastases in bone marrow (BM) could activate ROR1 CAR expression and lead to the elimination of normal ROR1+ BM stromal cells (57).

While antigen selection in synNotch receptor design typically adheres to general principles such as **coverage** (expression on a substantial fraction of tumor cells), **specificity** (expression on tumor cells but not on normal tissues), and **stability** of expression within tumor cells, additional criteria can be established based on the specific application of the synNotch receptor (142).​ For instance, if the objective is to localize synNotch-expressing cells to a particular site, leveraging anatomical cues becomes crucial. Similarly, when the aim is the controlled expression of a cytokine payload in a designated location, the same considerations apply. However, in the context of cancer, the complexity intensifies considerably, necessitating a cancer-specific framework for defining and refining antigen selection criteria. Overall, this idea can be regarded as a general principle, indicating that an antigen with higher specificity (even with heterogeneity) should be chosen as the prime antigen. In comparison, an antigen with greater coverage among cancer cells (even with lower specificity) should be chosen as the killing antigen. In this case, the safety and effectiveness of the synNotch system seem to be maximized based on the localized activation and trans-killing mechanism.

Of course, we should also point out that when normal and malignant tissues are geographically separate and not tightly co-located, synNotch CAR-T cells can Significantly expand the range of cell surface markers that can be safely targeted in cancer immunotherapy. Additionally, there is a less strict requirement that both target antigens be co-expressed on the same target cell because of the time delay between the activation of the synNotch receptor and the induction of CAR production. As a result, the synNotch method operates more like an “IF/THEN” logic gate than a rigid “AND” logic gate.

In the synNotch platform, the concept of avidity—which encompasses factors such as the expression level of the target antigen and the binding affinity of the recognition domain to the antigen—is pivotal for the system’s efficacy. These parameters collectively influence the sensitivity and specificity of T-cell responses.

For instance, increasing CAR affinity is one approach in this context; however, excessive affinity may lead to severe toxicity. In a study by Moghimi et al., a CAR-T cell targeting the GD2 antigen was produced using a high-affinity scFv (58). Despite the increased effectiveness of this CAR-T cell in mouse models of neuroblastoma compared to its lower-affinity counterpart, the former caused neurotoxicity and death. To address this issue, researchers created a synNotch CAR-T cell that used B7H3 as a gate to express the high-affinity GD2-targeting CAR construct. This type of synNotch CAR-T cell maintained effectiveness in eliminating neuroblastoma cells without causing any neurotoxicity. Therefore, synNotch CAR-T cells enable the use of high-affinity scFvs without the previous issues.

Or a different study engineered T-cell circuits that can detect varying antigen expression levels, enabling them to differentiate normal cells from cancerous ones based on antigen density (130).

While the significance of avidity in synNotch receptor design is recognized, further research is needed to define specific rules and thresholds for antigen expression levels and receptor binding affinities to optimize therapeutic outcomes.

Additionally, the metabolic state of synNotch CAR-T cells indicates potential advantages. The success of 4-1BB CAR-T cells is partly attributed to their enhanced fitness through oxidative phosphorylation, which generates ATP and improves persistence, demonstrating that metabolic preference (glycolytic vs. oxidative) significantly influences T-cell fate. SynNotch CAR-T cells, after eliminating target cells, exhibited an oxygen consumption rate similar to that of normal T cells, indicating that these gated T cells can revert to their naïve metabolic state. This improved metabolic flexibility and reprogramming towards oxidative phosphorylation support the idea that gated CAR-T cells have an expansion potential comparable to unmanipulated naïve T cells (58).

One prominent avenue in immunotherapy is immune checkpoint inhibition. Immune checkpoint inhibitors (ICIs) target regulatory receptors on T-lymphocytes, restoring T-cell function to enhance anti-tumor immunity (143). Under normal conditions, immune checkpoints balance pro- and anti-inflammatory signals (144), preventing excessive immune activation. These regulatory pathways include inhibitory and stimulatory mechanisms that influence immune cell activity (145). Several FDA-approved monoclonal antibodies block inhibitory immune checkpoints (146), and their combination with CAR T-cell therapy has been explored as a strategy to enhance therapeutic efficacy (147). Furthermore, ICIs can be directly incorporated into CAR T-cell designs to improve their functionality (148–150). Given the modular nature of synNotch receptors, ICIs could also be integrated into synNotch CAR T-cell designs. This approach would enable conditional immune checkpoint inhibition in response to tumor-specific signals, thereby mitigating systemic immune-related adverse effects while enhancing localized T-cell responses. Despite the therapeutic promise, there are currently no dedicated studies exploring synNotch CAR T-cell therapy specifically targeting immune checkpoints.

The Notch receptor’s ability to create new signaling pathways in target cells by not interfering with other downstream signaling pathways is a major advantage. Because of this benefit, it has been used in fields other than immunotherapy, such as tissue engineering and developmental biology. Subsequent investigations ought to concentrate on adapting this platform to additional immune cells, like macrophages, and assessing its efficacy and efficiency in these cells. Important research topics will also include examining immune cell fatigue and memory cell retention utilizing this platform.

Overall, while the current limitations of synNotch technology pose significant challenges, its potential to revolutionize targeted cancer therapies continues to drive research toward overcoming these barriers and optimizing clinical applications.

## Glossary

AAV: Adeno-associated virus
ADAM: A Disintegrin and Metalloprotease
ABD: Antigen-binding domain
AFP: Alpha-fetoprotein
ALPPL2: Alkaline phosphatase placental-like 2
BG: Benzylguanine
BITEs: Bi-specific T-cell Engagers
BM: Bone Marrow
CAR-T Cell: Chimeric antigen receptor T cell
CNS: Central nervous system
CRS: Cytokine Release Syndrome
EGFR: Epidermal Growth Factor Receptor
EGFRvIII: Epidermal growth factor receptor splice variant III
EMT: Epithelial-mesenchymal transition
EphA2: Ephrin type A receptor 2
FITC: Fluorescein isothiocyanate
HER2: Human epidermal growth factor receptor 2
HLA-A2: Human leukocyte antigen A2
IL13Rα2: Interleukin 13 receptor α2
iCARs: Inhibitory CAR-T cells
ITIM: Immunoreceptor tyrosine-based inhibitory motif
MART1: Melanoma antigen recognized by T-cells 1
MCAM: Melanoma Cell Adhesion Molecule
MHC: Major histocompatibility complex
MGMT: O-6-methylguanine-DNA methyltransferase
MMP: Matrix metalloproteinase
MOG: Myelin oligodendrocyte glycoprotein
mCD19: Murine CD19
OTOT: On-target off-tumor reaction
RIP: Regulated intramembrane proteolysis
scFv: Single-chain variable fragment
scid: Severe combined immunodeficiency
SFgfp: Superfolder green fluorescent protein
SNIPRs: Synthetic intramembrane proteolysis receptor
synNotch: Synthetic Notch
TAA: Tumor-Associated Antigen
TCR: T cell receptor
tBID: Truncated BH3-interacting domain death agonist
tCD19: Truncated form of CD19
tTa: Tetracycline transactivator
TME: Tumor microenvironment
TRE: Tetracycline response element
TSA: Tumor-specific antigens
UAS: Upstream Activation Sequence
VHH: Heavy-chain-only camelid antibodies
ICI: Immune checkpoint inhibitors

## Glossary of specialized terms and abbreviations

◼ A

AAV (Adeno-Associated Virus) – A small, non-pathogenic virus widely used as a gene delivery vector in gene therapy and CAR-T cell engineering.

ADAM (A Disintegrin and Metalloprotease) – A family of enzymes that play a role in protein cleavage, including the activation of Notch receptors, affecting cell signaling and immune modulation.

AFP (Alpha-Fetoprotein) – A tumor marker primarily found in liver cancer and germ cell tumors, sometimes targeted in immunotherapy.

ALPPL2 (Alkaline Phosphatase Placental-Like 2) – A tumor-associated antigen used as a marker in cancer research and immunotherapy.

AND Gate – A Boolean logic circuit in synthetic biology where two or more signals must be present to trigger a cellular response, improving specificity in CAR-T therapy.

◼ B

BG (Benzylguanine) – A chemical group used in bioengineering to modify antibodies or receptors for experimental applications.

BITEs (Bi-Specific T-Cell Engagers) – A class of engineered antibodies that simultaneously bind to a tumor antigen and a T-cell receptor, enhancing immune cell targeting.

BM (Bone Marrow) – The tissue responsible for producing blood cells, including immune cells essential for CAR-T therapy.

◼ C

CAR-T Cell (Chimeric Antigen Receptor T Cell) – Genetically engineered T cells that express artificial receptors to specifically recognize and attack cancer cells.

CNS (Central Nervous System) – Comprising the brain and spinal cord, often a challenging site for CAR-T therapy due to the blood-brain barrier.

CRS (Cytokine Release Syndrome) – A severe immune response triggered by excessive cytokine production in CAR-T cell therapy.

◼ E

EGFR (Epidermal Growth Factor Receptor) – A transmembrane protein involved in cell growth and commonly overexpressed in certain cancers.

EGFRvIII (Epidermal Growth Factor Receptor Splice Variant III) – A mutant form of EGFR found in glioblastomas, making it a selective target for immunotherapy.

EMT (Epithelial-Mesenchymal Transition) – A biological process in which epithelial cells gain migratory properties, often associated with cancer metastasis.

EphA2 (Ephrin Type A Receptor 2) – A receptor tyrosine kinase implicated in cancer progression and targeted in immunotherapy.

◼ F

FITC (Fluorescein Isothiocyanate) – A fluorescent dye used in biological assays for protein and cell labeling.

◼ H

HER2 (Human Epidermal Growth Factor Receptor 2) – A receptor frequently overexpressed in breast and other cancers, serving as a CAR-T therapy target.

HLA-A2 (Human Leukocyte Antigen A2) – A key molecule in antigen presentation, crucial for T-cell recognition in cancer immunotherapy.

◼ I

IL13Rα2 (Interleukin 13 Receptor Alpha 2) – A tumor-associated antigen frequently found in glioblastoma, targeted in CAR-T therapy.

iCARs (Inhibitory CARs) – CAR constructs designed to suppress T-cell activation upon detecting non-cancerous tissues, reducing off-target effects.

ITIM (Immunoreceptor Tyrosine-Based Inhibitory Motif) – A sequence in immune receptors that transmits inhibitory signals, dampening immune activation.

Immune Checkpoint Inhibitors (ICIs) – Monoclonal antibodies or other molecules that block inhibitory checkpoint proteins, enhancing T-cell responses against cancer.

◼ L

Logic Gates (Boolean Logic in Synthetic Biology) – Computational-like systems that enable precise control of gene expression in CAR-T therapy. Includes:

AND Gate – Requires two antigens for activation, enhancing specificity.

OR Gate – Activates if at least one of the target antigens is present.

NOT Gate – Suppresses activation when a certain antigen is present.

AND-NOT Gate – Requires one antigen while excluding another for activation.

Multi-Input AND Gate – A higher-order logic circuit requiring multiple antigen signals to trigger activation.

◼ M

MART1 (Melanoma Antigen Recognized by T Cells 1) – A melanoma-associated antigen recognized by T-cell therapies.

MCAM (Melanoma Cell Adhesion Molecule) – A cell surface protein involved in melanoma progression and metastasis.

MHC (Major Histocompatibility Complex) – A set of proteins crucial for antigen presentation to T cells.

MGMT (O-6-Methylguanine-DNA Methyltransferase) – A DNA repair enzyme linked to chemotherapy resistance.

MMP (Matrix Metalloproteinase) – A group of enzymes that degrade extracellular matrix, facilitating tumor invasion.

MOG (Myelin Oligodendrocyte Glycoprotein) – A CNS antigen relevant in autoimmunity and neuro-oncology.

mCD19 (Murine CD19) – A mouse version of the CD19 antigen, used in preclinical CAR-T research.

◼ N

NOT Gate – A Boolean logic circuit that suppresses activation when a specific antigen is detected.

◼ O

OTOT (On-Target Off-Tumor Reaction) – An unintended effect in CAR-T therapy where normal tissues expressing the target antigen are attacked.

◼ R

RIP (Regulated Intramembrane Proteolysis) – A signaling mechanism that processes transmembrane proteins like Notch, influencing gene expression.

◼ S

scFv (Single-Chain Variable Fragment) – A small antibody fragment used in CAR constructs for antigen recognition.

scid (Severe Combined Immunodeficiency) – A genetic disorder causing immune deficiency, often studied in mouse models for CAR-T therapy.

SFgfp (Superfolder Green Fluorescent Protein) – A highly stable fluorescent protein used in research applications.

SNIPRs (Synthetic Intramembrane Proteolysis Receptors) – Synthetic receptors designed to regulate cell signaling and function in therapeutic applications.

SynNotch (Synthetic Notch) – An engineered receptor inspired by Notch signaling, allowing for precise gene regulation in CAR-T therapies.

◼ T

TAA (Tumor-Associated Antigen) – An antigen expressed on both normal and tumor cells, making targeted therapy challenging.

TCR (T Cell Receptor) – The receptor on T cells that detects antigen-MHC complexes.

tBID (Truncated BH3-Interacting Domain Death Agonist) – A pro-apoptotic protein that mediates programmed cell death.

tCD19 (Truncated CD19) – A modified form of CD19 used in synthetic biology applications.

tTa (Tetracycline Transactivator) – A regulatory protein used in inducible gene expression systems.

TME (Tumor Microenvironment) – The cellular and molecular surroundings of a tumor, influencing immune responses and treatment efficacy.
